# Do statins reduce mortality in older people? Findings from a longitudinal study using primary care records

**DOI:** 10.1136/fmch-2020-000780

**Published:** 2021-05-24

**Authors:** Lisanne Andra Gitsels, Ilyas Bakbergenuly, Nicholas Steel, Elena Kulinskaya

**Affiliations:** 1 Population, Policy and Practice Research and Teaching Department, Great Ormond Street Institute of Child Health, University College London, London, UK; 2 School of Computing Sciences, University of East Anglia, Norwich, UK; 3 Norwich Medical School, University of East Anglia, Norwich, UK

**Keywords:** cardiovascular diseases, epidemiology, health records, personal

## Abstract

**Objective:**

Assess whether statins reduce mortality in the general population aged 60 years and above.

**Design:**

Retrospective cohort study.

**Setting:**

Primary care practices contributing to The Health Improvement Network database, England and Wales, 1990–2017.

**Participants:**

Cohort who turned age 60 between 1990 and 2000 with no previous cardiovascular disease or statin prescription and followed up until 2017.

**Results:**

Current statin prescription was associated with a significant reduction in all-cause mortality from age 65 years onward, with greater reductions seen at older ages. The adjusted HRs of mortality associated with statin prescription at ages 65, 70, 75, 80 and 85 years were 0.76 (95% CI 0.71 to 0.81), 0.71 (95% CI 0.68 to 0.75), 0.68 (95% CI 0.65 to 0.72), 0.63 (95% CI 0.53 to 0.73) and 0.54 (95% CI 0.33 to 0.92), respectively. The adjusted HRs did not vary by sex or cardiac risk.

**Conclusions:**

Using regularly updated clinical information on sequential treatment decisions in older people, mortality predictions were updated every 6 months until age 85 years in a combined primary and secondary prevention population. The consistent mortality reduction of statins from age 65 years onward supports their use where clinically indicated at age 75 and older, where there has been particular uncertainty of the benefits.

**Key points**

**Question**

- Current guidelines on the prevention of cardiovascular disease are cautious with recommending statin therapy to people aged 75 years or older due to the limited evidence from clinical trials of the overall benefit in this group. This study aimed to clarify whether current statin prescription reduces mortality in the older population, with updated survival predictions every six months from age 60 to 85 years reflecting fluid clinical practice.

**Finding**

- Current statin prescription was associated with significant absolute and relative reduction in mortality at older ages, including those aged 75 years or older, irrespective of sex and cardiac risk.

**Meaning**

- This supports the use of statins in those aged 75 years or older, where clinically indicated and after discussion of the potential risks and benefits with the patient.

## Introduction

Cardiovascular disease (CVD) is the biggest cause of death from non-communicable disease in the world.^1^ Clinical guidelines internationally recommend statin therapy for both primary and secondary prevention of CVD, making statins one of the most frequently prescribed drugs in industrialised countries.^2–5^ Yet uncertainty remains about the benefits of statin therapy in older people, particularly those aged 75 years or older who do not have a history of CVD, due to relatively few older people being included in randomised controlled trials (RCTs).^6–8^ In 2019, the Cholesterol Treatment Trialists’ Collaboration published a meta-analysis of 28 randomised trials of statin therapy in older people. They found a trend towards smaller relative reductions in cardiac events and vascular mortality with increasing age, and no effect on non-vascular mortality.^9^ Their risk ratios, however, were inappropriately presented as per mmol/L reduction in low-density lipoprotein (LDL, ‘bad cholesterol’), making it difficult to compare with findings of other studies, and their data have not been made available for scrutiny. A recent meta-analysis of RCTs and a contemporary cohort showed the efficacy of LDL lowering treatment for primary prevention of cardiovascular events for patients aged 75 or older.^10 11^ However, another study found that low and high levels of LDL was associated with increased risk of all-cause mortality, questioning the safety of cholesterol-lowering.^12^ The persistent uncertainty about the benefits and risks of statins in those aged over 75 is being addressed by trials including the STAREE trial, which is examining whether statin therapy will reduce all-cause mortality among healthy older people (≥70 years), with an estimated study completion date of December 2023.^13^

A limitation of RCTs, however, is that they tend to be of relatively short duration, with the CTT trials having a median follow-up of almost 5 years^9^ and STAREE planned to have an average of 5-year treatment period,^13^ whereas statins are usually prescribed for life. This means that still little is known about the long-term overall effects of statin therapy. Furthermore, in the clinical practice, patients are not fixed on a certain treatment regime as during an RCT but instead sequential treatment decisions are made in managing their changing cardiac risk.^14^

We previously studied the long-term survival benefit of a history of statin prescription for primary prevention of CVD, without taking into account time-varying covariates, and found a significant reduction in all-cause mortality from age 65 onward.^15^ Whereas a recent study showed that current but not former use of statins for primary prevention of CVD is effective in people aged 75 years and older.^16^ Our study assessed whether current statin prescription for primary and secondary prevention of CVD reduces mortality in the general population, with updated survival predictions every 6 months from age 60 to 85 years reflecting fluid clinical practice.

## Methods

### Study design

A retrospective cohort study was designed using EHR from The Health Improvement Network (THIN) primary care database. THIN records are broadly representative of the UK population prevalence of medical conditions and mortality rates when adjusted for sex, age and deprivation.^17 18^ The study recruitment period was from January 1990 to December 2000. The start of the study period was chosen to be approximately 2 years after the first commercial statin was approved by health authorities,^19^ to ensure that the therapy would be available to the study population. The follow-up period was to January 2017 with medical history updated every 6 months.

The cohort included patients who, at baseline, were aged 60, residential in England or Wales and had no medical history of statin therapy nor CVD as defined by QRISK researchers (coronary heart disease and cerebrovascular disease, but not peripheral vascular disease).^20^ This allowed to study new statins users for primary prevention of CVD and, as a proportion of patients was expected to develop CVD and be prescribed statins during follow-up, also secondary prevention. As clinical guidelines on statin therapy and its uptake in clinical practice changed rapidly after its introduction to the world market, we distinguished two birth cohorts of patients born in 1930–1935 and 1936–40.^21^

### Covariates

The outcome was time to all-cause mortality, which encompasses both benefits (eg, reduction in CVD deaths) and adverse events (eg, increased competing mortality). The exposure was statin therapy, which was measured as a current prescription (yes/no).

Covariates adjusted for were sex, birth cohort, cardiac risk, chronic kidney disease (CKD), diabetes, treated hypertension, hypercholesterolaemia, body mass index (BMI), aspirin prescription, alcohol consumption, smoking, Townsend deprivation quintile and general practice (online supplemental table S1).^15 22–25^ Cardiac risk was categorised as low, medium and high corresponding to a QRISK2 score of <20%, 20%–39% and ≥40% or CVD diagnosis, respectively. As in our previous paper,^15^ QRISK2 scores were calculated using the information on age, sex, Townsend deprivation score, CKD, diabetes, systolic blood pressure (SBP), treated hypertension, hypercholesterolaemia, BMI and smoking.

10.1136/fmch-2020-000780.supp1 Supplementary data

There were missing values in SBP, BMI, alcohol consumption and smoking, which were less common in women (up to age 75), at older ages, in diagnosed or prescribed patients, and later calendar years (online supplemental table S2). At baseline, 63% of the sample had at least one missing item, which was dealt with by multiple imputations of 10 datasets^26 27^ (online supplemental figure S1). Missing data after baseline were dealt with by last observation carried forward.^28^

### Statistical analyses

A Cox regression model was fitted to estimate the hazard of all-cause mortality associated with current statin prescription. This was done for follow-up times of 5, 10 and 25 years and fitted every 6 months from age 60 to 85 years, creating a sliding time window. This technique is called landmarking where the latest medical history is used at the new time point (‘landmark’) conditional on survival to that point, thereby allowing for time-dependent covariates and mortality effects, and predictions beyond the study period.^28^ The advantage of this technique over time-dependent Cox regression is its transparency of what is being compared at each time point while the inferences of the latter are only valid if the value of the time-dependent covariate is observed for all subjects at all event time points.^29^

The landmarking modelling process is explained in the online supplemental file 1 and more detailed in our methodology paper.^30^ We tested for interactions of current statin prescription with sex, birth cohort and cardiac risk, which were included if significant at p<0.05. The model was assessed on the proportional hazards assumption and discrimination using the concordance index.^31^ The sensitivity analyses included (1) fitting the model while restricting the controls to only patients who were never prescribed statins, and (2) fitting the model on the subset of participants without missing data (‘complete-case analysis’). All statistical analyses were carried out in R V.3.5.0, except for the QRISK2 score calculation in JAVA V.10.

The 10-year HRs were translated to absolute and relative risk reduction (ARR and RRR) at key ages by various risk profiles to inform decision making in clinical guidelines.^32^ The profiles were stratified by sex, birth cohort, cardiac risk, health status and deprivation. The incidence of diabetes in patients previously on or off statins prescription was calculated at each landmark to assess the potential risk of diabetes resulting from statin therapy.

## Results

### Study population

The study population comprised 110 243 patients ages 60 years with 38% entering the study in 1990–1995 (born in 1930–1935) and the remaining 62% in 1996–2000 (born in 1936–1940) (table 1). The median follow-up was 16.6 years and maximum for 27 years, with 78 728 (71%) participating at age 75 (online supplemental table S3). During the follow-up, the cardiac risk of the study population increased, largely driven by age (online supplemental table S4). The overall mortality rate per year during follow-up was 120 per 10 000 people, where death was observed in 20% of the study population by the end of the study period.

**Table 1:** Characteristics of the study population

		QRISK2 <20%*		QRISK2=20%–39%*	
		Women (%)	Men (%)	Women (%)	Men (%)
Cohort size		59 082	48 836	314	2011
Person-years of follow-up (median)		1 002 627 (17.0)	789 677 (16.2)	4291 (13.7)	28 900 (14.4)
Deaths during follow-up		10 152 (17%)	10 743 (22%)	152 (48%)	814 (40%)
Transferred during follow-up		15 410 (26%)	13 401 (27%)	70 (22%)	422 (21%)
Birth cohort	1930–35	23 139 (39%)	18 224 (37%)	92 (29%)	594 (30%)
	1936–40	35 943 (61%)	30 612 (63%)	222 (71%)	1417 (70%)
Deprivation	First quintile	15 481 (26%)	13 681 (28%)	12 (4%)	315 (16%)
	Second quintile	13 652 (23%)	11 376 (23%)	22 (7%)	342 (17%)
	Third quintile	12 149 (21%)	9775 (20%)	37 (12%)	388 (19%)
	Fourth quintile	10 310 (17%)	8007 (16%)	80 (25%)	442 (22%)
	Fifth quintile (most deprived)	7492 (13%)	5996 (12%)	162 (52%)	525 (26%)
CKD	No	59 073 (100%)	48 835 (100%)	312 (99%)	2004 (100%)
	Diagnosed	9 (0%)	1 (0%)	2 (1%)	7 (0%)
Diabetes	No	57 952 (98%)	47 973 (98%)	67 (21%)	1085 (54%)
	Diagnosed	1130 (2%)	863 (2%)	247 (79%)	926 (46%)
Hypertension	No	44 411 (75%)	38 829 (80%)	54 (17%)	406 (20%)
	Diagnosed and treated	6443 (11%)	3200 (7%)	204 (65%)	1128 (56%)
	Diagnosed and not treated	8229 (14%)	6807 (14%)	55 (18%)	477 (24%)
SBP	Mean (SD) in mm Hg	135.3 (17.33)	133.71 (16.61)	159.42 (20.11)	153.49 (17.72)
Aspirin	No	58 490 (99%)	48 369 (99%)	293 (93%)	1918 (95%)
	Prescribed	592 (1%)	467 (1%)	21 (7%)	93 (5%)
HCL	No	55 966 (95%)	46 127 (94%)	243 (78%)	1638 (81%)
	Diagnosed	3117 (5%)	2709 (6%)	71 (22%)	373 (19%)
BMI	Healthy weight	23 281 (39%)	18 205 (37%)	50 (16%)	527 (26%)
	Overweight	22 739 (38%)	21 520 (44%)	98 (31%)	834 (41%)
	Obese	13 063 (22%)	9111 (19%)	167 (53%)	651 (32%)
Alcohol	Non-current	30 922 (52%)	17 306 (35%)	136 (43%)	429 (21%)
	Current	28 160 (48%)	31 530 (65%)	178 (57%)	1582 (79%)
Smoking	Non	47 000 (80%)	34 717 (71%)	63 (20%)	412 (20%)
	Ex	3984 (7%)	5644 (12%)	45 (14%)	231 (11%)
	Current	8098 (14%)	8476 (17%)	206 (66%)	1369 (68%)

*Mean across ten imputed datasets.

BMI, body mass index; CKD, chronic kidney disease; HCL, hypercholesterolaemia; SBP, systolic blood pressure.

**Figure 1:**
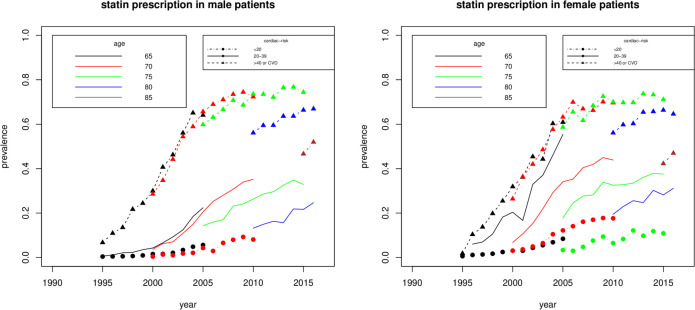
Proportion of current statin prescription by cardiac risk, sex and age during the study period.

The proportion of current statin prescription increased greatly between 1995 and 2010 and roughly stabilised afterwards, with differences seen by cardiac risk, sex, and age (figure 1). In 2015, current statin prescription in 75 year olds at low, medium and high cardiac risk was 10%, 35% and 75%, respectively.

The cumulative years of statin prescription increased during the follow-up and were greater in the younger birth cohort (online supplemental figure S2). The median (IQR) statin exposure in those with a current prescription at age 75 and born in 1930–1935 or 1936–1940 was 4 (2-7) years or 6 (3-9) years, respectively. Approximately 90% of patients with a current statin prescription adhered at least 75% of follow-up time (S3 Figure). By the end of the study period, 51% of the study population were never prescribed statins, 41% were prescribed at some age and stayed on statins, 6% were prescribed statins and subsequently stopped statins permanently and 2% initially stopped statins but came back on, including 0.5% who had four or more switches. The cumulative years of statin prescription in patients with no current statin prescription at a landmark also showed minimal crossover of treatment (online supplemental figure S4).

### All-cause mortality reductions

**Figure 2:**
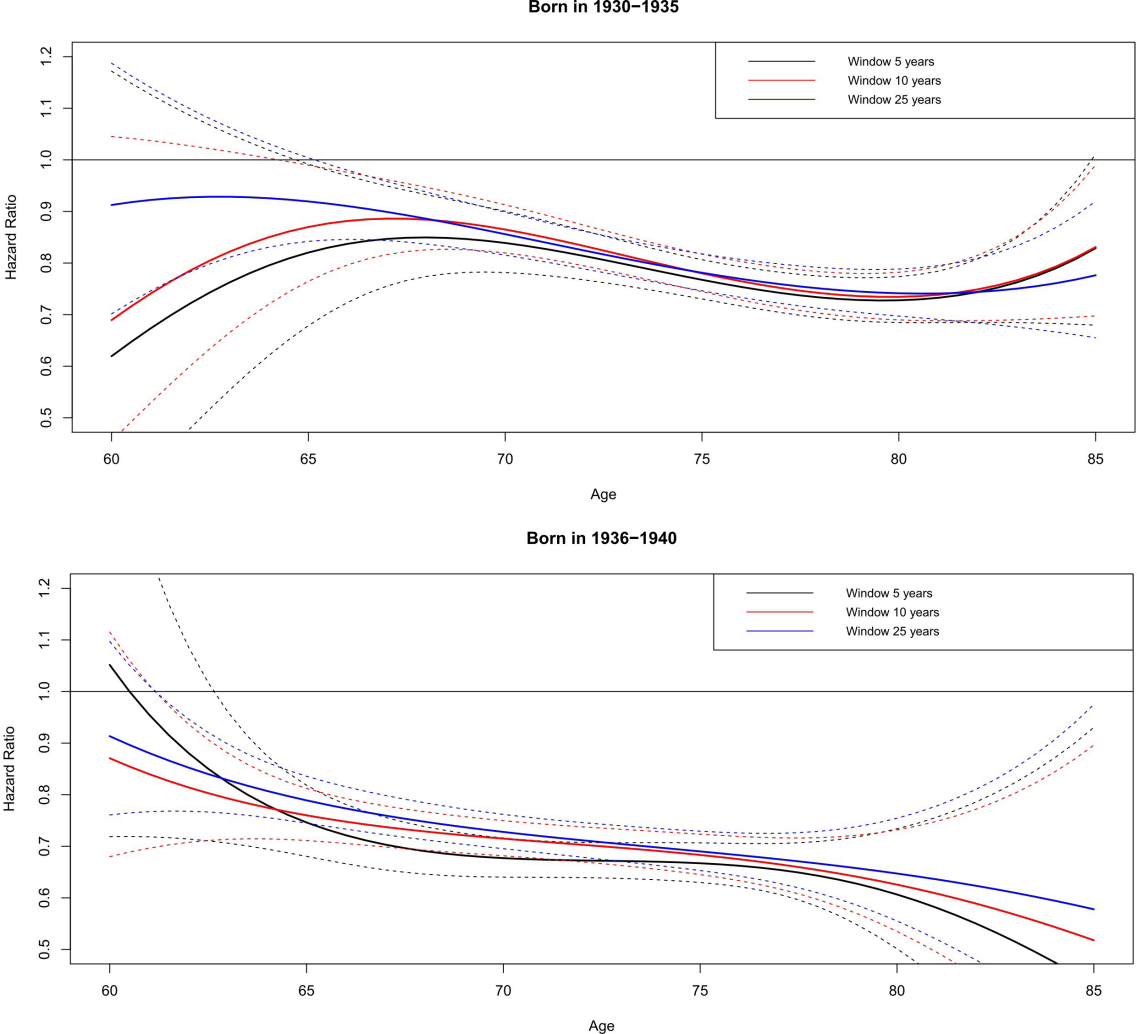
Adjusted HRs of all-cause mortality associated with current statin prescription. Adjusted for sex, cardiac risk, deprivation, chronic kidney disease, diabetes, hypertension, hypercholesterolaemia, aspirin, body mass index, alcohol consumption and smoking.

Current statins prescription was associated with a significant reduction in all-cause mortality from age 65 years onward, with greater reductions seen at older ages and in the youngest birth cohort (figure 2). Compared with no current statin prescription, a prescription at key ages 65, 70, 75, 80 and 85 in for the youngest birth cohort (born in 1936–1940) was associated with 10 years HRs of 0.76 (95% CI 0.71 to 0.81), 0.71 (95% CI 0.68 to 0.75), 0.68 (95% CI 0.65 to 0.72), 0.63 (95% CI 0.53 to 0.73) and 0.54 (95% CI 0.33 to 0.92) respectively. Similarly for the oldest birth cohort (born in 1930–1935), current statin prescription at key ages 65, 70, 75, 80 and 85 was associated with 10 years HRs of 0.87 (95% CI 0.76 to 0.99), 0.86 (95% CI 0.82 to 0.91), 0.78 (95% CI 0.74 to 0.82), 0.73 (95% CI 0.69 to 0.78) and 0.84 (95% CI 0.69 to 1.03) respectively. Compared with the 10 years HRs, the 5 years and 25 years ones differed by no more than 5 percentage points and the differences were not significantly different (figure 2 and online supplemental table S5). There was no significant interaction between current statin prescription and sex or cardiac risk.

The mortality probabilities and ARR and RRR associated with current statin prescription are presented in table 2. The ARR increased with increasing age and cardiac risk, and was higher in men and those with poor health status. For example, at age 80 years, statin prescription may prevent an average of 164 or 171 deaths per 1000 men with poor health status at medium or high cardiac risk, respectively. In comparison, an average of 82 or 101 deaths may be prevented for every 1000 men prescribed with the best health status at medium or high cardiac risk.

**Table 2:** 10-year mortality probabilities for non-statin prescription, and the absolute risk reduction (ARR) and relative risk reduction (RRR) in all-cause mortality associated with current statin prescription by risk profiles, presented in percentages

Men		No statins	Statins	Statins
**Age**	QRISK2	Deaths	Born 1930–35 ARR/RRR	Born 1936–40 ARR/RRR
**Profile A**				
65	<20	7%	0.86/12.62	1.60/23.36
	20–40	8%	0.96/12.57	1.77/23.28
	≥40 or CVD	10%	1.24/12.43	2.29/23.06
70	<20	13%	1.62/12.73	3.46/27.14
	20–40	14%	1.79/12.63	3.83/26.98
	≥40 or CVD	20%	2.40/12.27	5.14/26.34
75	<20	NA	NA	NA
	20–40	25%	4.96/19.56	7.25/28.58
	≥40 or CVD	34%	6.34/18.56	9.32/27.29
80	<20	NA	NA	NA
	20–40	24%	5.74/23.95	8.21/34.27
	≥40 or CVD	30%	7.01/23.15	10.08/33.28
**Profile B**				
65	<20	37%	3.93/10.53	7.44/19.93
	20–40	41%	4.17/10.26	7.91/19.48
	≥40 or CVD	50%	4.71/9.46	9.03/18.13
70	<20	49%	4.86/9.91	10.80/22.04
	20–40	53%	5.05/9.52	11.30/21.30
	≥40 or CVD	66%	5.33/8.11	12.24/18.62
75	<20	NA	NA	NA
	20–40	69%	9.11/13.20	13.93/20.18
	≥40 or CVD	81%	8.35/10.28	13.12/16.15
80	<20	NA	NA	NA
	20–40	60%	11.04/18.29	16.41/27.18
	≥40 or CVD	70%	11.30/16.04	17.09/24.26
**Women**		**No statins**	**Statins**	**Statins**
**Age**	QRISK2	Deaths	Born 1930–35 ARR/RRR	Born 1936–40 ARR/RRR
**Profile A**				
65	<20	5%	0.67/12.71	1.23/12.37
	20–40	6%	0.74/12.68	1.37/12.34
	≥40 or CVD	8%	0.96/12.57	1.78/12.25
70	<20	10%	1.29/12.90	2.74/16.71
	20–40	11%	1.43/12.83	3.04/16.63
	≥40 or CVD	15%	1.93/12.55	4.14/16.33
75	<20	20%	4.03/20.12	5.86/11.49
	20–40	20%	4.07/20.10	5.93/11.48
	≥40 or CVD	28%	5.33/19.31	7.80/11.09
80	<20	NA	NA	NA
	20–40	19%	4.69/24.52	6.69/13.84
	≥40 or CVD	24%	5.83/23.89	8.35/13.54
**Profile B**				
65	<20	30%	3.32/11.09	6.24/10.99
	20–40	33%	3.56/10.88	6.71/10.81
	≥40 or CVD	41%	4.18/10.25	7.94/10.26
70	<20	40%	4.32/10.67	9.50/14.30
	20–40	44%	4.57/10.36	10.10/13.95
	≥40 or CVD	56%	5.17/9.19	11.63/12.66
75	<20	59%	8.89/15.04	13.40/8.97
	20–40	60%	8.91/14.96	13.44/8.93
	≥40 or CVD	73%	9.03/12.44	13.90/7.66
80	<20	NA	NA	NA
	20–40	51%	10.25/20.00	15.04/11.69
	≥40 or CVD	61%	11.09/18.13	16.50/10.80

Profile A: healthiest and wealthiest profile included non-smokers without a diagnosis of diabetes, hypertension, hypercholesterolemia, or chronic kidney disease, and resident in the least deprived area. Profile B: least healthy and wealthy profile included smokers with a diagnosis of diabetes, untreated hypertension, and resident in the most deprived area.

CVD, cardiovascular disease; NA, not available.

Regarding the main side effect of statins, the unadjusted incidence of diabetes during the study period was initially almost three times as high in participants with prior statin prescription (9 in 1000) compared with those without (3 in 1000), but by age 85 the rate was the same (4 in 1000) (online supplemental figure S5). The difference in rates was not significant in men at most ages and not significant in women from age 68 years onward.

The sensitivity analysis that compared participants currently prescribed statins to those never prescribed, found an opposite trend of decreasing mortality reductions by increasing age; starting with lower HRs at age 60 years and converging to the same predicted reductions by age 80 years, with no significant difference between the main and sensitivity analyses from age 75 years onward (online supplemental figure S6). In contrast, the complete case analysis showed very similar results to the main analysis, with overlapping confidence intervals of the HRs associated with current statin prescription (online supplemental table S6).

## Discussion

This study predicted the 5 years, 10 years and 25 years hazards of all-cause mortality associated with current statin prescription over 25 years in an older population of primary and secondary prevention of CVD in England and Wales. This was achieved by creating a so-called sliding window using the latest medical history known at a time point to update the HRs, reflecting the sequential treatment decisions made in primary care. Current statin prescription was associated with a significant reduction in mortality from age 65 years onward. The reduction increased by age with at least an HR of 0.68 in people aged 75 or older. The sensitivity analysis of comparing benefits of current statin prescription to no prescription ever found greater reductions in mortality at younger ages yet similar reductions in people aged 75 or older. Both analyses are useful in routine clinical practice as the main analysis answers the question what the overall benefit is of statin uptake now compared with potential future uptake, whereas the sensitivity analysis answers the question what the overall benefit is of statin uptake compared with never uptake.

The study also found that the mortality reductions associated with current statin prescription differed by birth cohort, where people born in later years had greater benefits. This could be explained by the changing availability and recommended dosages of statin types resulting in more effective treatment over time, such as the withdrawal of cerivastatin from the world market in 2001 due to harmful effects.^21^ There were no other mortality differences associated with statin therapy, including no interaction with sex and cardiac risk. This translated to similar relative gains from statins across risk levels and profiles, yet the greatest absolute gains were seen in those at greatest risk.

Individuals taking statins could experience side effects of which diabetes could potentially have long-term consequences for health.^33^ The most important outcome, however, is overall survival, which directly tallies up the benefits and harms of treatment. This is especially true for older people, where preventative treatments should not be focused on altering the cause of death but on prolonging life.^6^ This study showed that the incidence rate of diabetes was higher among participants with prior statin prescription compared with those without, however, current statin prescription was associated with mortality reductions irrespective of diabetes diagnosis and from age 70 years onward participants with a current statin prescription and diagnosis of diabetes had better overall survival prospects than those without a prescription and no diagnosis.

This study was designed to address the current gap in clinical guidelines internationally on the uptake of statin therapy for primary and secondary prevention of CVD in older people, particularly those aged 75 years or older who do not have a history of CVD. Based on 1.8 million person-years of routinely collected primary care data, this study estimated and updated biyearly the long-term survival prospects associated with current statin therapy by incorporating participants’ time-varying prescription, cardiac risk and health status from age 60 to 85 years. The resulting dynamic risk model reflects clinical practice where sequential treatment decisions are made based on the patient’s health status over the life course and thereby predicts future health status more accurately compared with a static risk model.^34^

In this study, the survival effects of statin therapy were approximated by current statin prescription. This intention-to-treat analysis, however, could give conservative treatment effects when there is lower actual uptake than observed prescription, low adherence in the treatment group and/or initiation of treatment in the control group.^35^ The purpose of the landmark approach for survival models is to capture the development over time including changing treatment^28^ and our study showed minimal crossover of treatment arms. In the case of conservative treatment effects, it means that statin therapy is associated with even greater reductions in mortality as was estimated in our sensitivity analysis. On the other hand, there could be a healthy user bias in which individuals who are prescribed statins have a healthier lifestyle compared with those who do not and in turn better survival prospects.^36^ This effect is well documented with statins in observational studies and would result in overestimated treatment effects.^36^ However, even propensity score matched analyses that deals with healthy user bias have reported significant reductions in mortality associated with statin therapy in people aged 75 years and older.^37^

Another limitation of an observational study is the possibility of confounding by indication. We attempted to minimise this by adjusting the regression analyses for known confounders of the effect of statin therapy associated with survival prospects, including cardiac risk and related comorbidities and treatments. Finally, although there were missing data, they were appropriately dealt with by multiple imputations and had very similar results as the complete case analysis.

Limited research exists on the overall benefit of statin in older people for long-term use. The influential CTT meta-analysis included from 19 out of 28 RCTs 8043 and 6449 participants with and without a history of CVD, respectively, who were older than 75 years and on average 78.8 years at baseline.^9^ The median follow-up of the RCTs was almost 5 years, but this included all ages and there was very likely shorter follow-up at the older ages. The current STAREE statin trial aims to have 18 000 participants aged 70 years or older without a history of CVD and follow them up for a 5-year treatment period.^13^ In contrast, our study included 15 761 and 62 967 participants with and without a history of CVD at age 75, and these numbers were 8457 and 22 710 at age 80. Our median follow-up was over 15 years, although this was almost 5 years at age 75, 3 years at age 80, and 1 year at age 85, nevertheless landmarking analysis allows to confidently predict survival prospects beyond the study period.^28^ The CTT meta-analysis included RCTs with strict inclusion criteria that limit the generalisability to the general population and fitted a static model with no updated statin exposure. Our modelling process with updated statin exposure and survival predictions are more sensitive to age differences and could be more useful in clinical practice, where patients come to clinicians at any age and multiple times. Furthermore, the CCT meta-analysis reported risk ratios as per mmol/L reduction in LDL (‘bad cholesterol’), making it difficult to compare results. Finally, they did not distinguish between statin therapy for primary or secondary prevention, whereas our study did and found that the extent of mortality reduction was not significantly different for primary and secondary prevention of CVD.

Other observational studies reported that statin therapy for primary prevention of CVD was associated with reductions in all-cause mortality in a population of aged 75 or older in the USA,^37^ in Korea,^16^ in the UK if at high cardiac risk,^15^ and in Spain if in presence of diabetes,^25^ and discontinuation of statins in 75 years is associated with increased risk of a first cardiac event.^38^ Our findings add to the evidence base that statins are not only beneficial at a static moment but can be initiated and continued at older ages with long-term survival benefits.

## Conclusions

Our study showed significant absolute and relative reductions in all-cause mortality associated with statins for primary and secondary prevention of CVD at all ages over 65 years, including those at age 75 or older, in routine clinical practice over 25 years. This supports the use of statins, where clinically indicated and after discussion of the potential risks and benefits with the patient, in those aged over 75 years.

## Data Availability

Data may be obtained from a third party and are not publicly available. For all interested researchers, THIN data are available via QuintilesIMS, subject to ethical approval of the THIN Scientific Review Committee and governance controls.
