# Supplementary material for: Do statins reduce mortality in older people? Findings from a longitudinal study using primary care records
Source: Fam Med Community Health. 2021 May 24;9(2):e000780. doi: 10.1136/fmch-2020-000780 (PMC8149437; doi:10.1136/fmch-2020-000780)

Supplementary file

Tables

S1 Table. Coding of medical history. [a]Latest reading at landmark age. [b]First category functioned as the reference group.

S2 Table. Characteristics of patients with complete and incomplete[a] health records. [a]Missing data in systolic blood pressure, body mass index, alcohol consumer status or smoking status.

S3 Table. Length of follow-up by cardiac risk and landmark age.

S4 Table. Cardiac risk by sex and landmark age. [a]Mean across ten imputed datasets.

S5 Table. Hazards of all-cause mortality from landmark model fitted on imputed datasets. [a]All hazard ratios were adjusted by the factors listed in this table except for 'statins - unadjusted'. [b]Prescription at landmark age.

S6 Table. Hazards of all-cause mortality from landmark model fitted on complete cases. [a]All hazard ratios are adjusted by the factors listed in this table except for 'statins - unadjusted'. [b]Prescription at landmark age.

Figures

S1 Figure. Distribution of known and imputed values of variables with missing data at baseline.

S2 Figure. Cumulative years[a] of statin therapy by the landmark age in patients with current statin prescription by year of birth. [a]Cumulative years of statin therapy was defined from first prescription until landmark *s* in patients with current statin prescription at landmark *s*.

S3 Figure. Proportion of high adherence[a] to statin therapy in patients with a current statin prescription by year of birth. [a]High adherence to statin therapy was defined as statin prescription at least 75% of the time from landmark *s* until end of follow-up in patients with a current statin prescription at *s*.

S4 Figure. Average cumulative years[a] of statin therapy by the landmark age in patients with no current statin prescription by year of birth. [a]Cumulative years of statin therapy was defined from first prescription until landmark *s-1* in patients with no current statin prescription at landmark *s* and was zero for patients who never had a statin prescription until landmark *s-1*.

S5 Figure. Incidence of diabetes in patients with or without prior statin prescription by the landmark age.

S6 Figure. Sensitivity analysis: hazards of all-cause mortality associated with current statin prescription excluding controls with future statin prescription fitted on imputed datasets.

Methods landmark analyses

Landmark analyses were carried out to dynamically predict the survival effects associated with statin therapy based on the latest medical history at the landmark, thereby allowing for time-dependent covariates and survival effects, and predictions at landmarks beyond the study period (1). Cox regression models were fitted to predict 5-, 10-, and 25-year survival associated with current statin prescription at each landmark (every six months) from age 60 to 85 years, resulting in 51 landmarks. This modelling process involved four stages. At stage one, a Cox model was fitted on complete cases at baseline age to inform the imputation model. The baseline analysis model included all covariates and tested for interactions of cardiac risk with sex and birth cohort using backward elimination. As none of the interactions was significant, the imputation model included only medical history without interactions. At stage two, Cox models were fitted on the imputed datasets at ages 65, 70, 75, 80 and 85 to inform the final landmark model. These models included all medical history and tested for interactions of statin prescription with sex, birth cohort and cardiac risk. The final landmark model included the medical history and the significant interaction between statin prescription and birth cohort. This interaction was defined as no statin prescription (reference level), statin prescription in patients born in 1930-35, and statin prescription in patients born in 1936-40. At stage three, an unadjusted Cox landmark model for current statin prescription and the final, fully adjusted, Cox landmark model was fitted on the imputed datasets. The landmark effect was smoothed with an integrated pseudo partial log-likelihood (IPL*) (2). At stage four, the results of the previous stage were pooled using Rubin rules (3). While the models in stages one and two were multilevel on general practice, the landmark models in stage three could only adjust for within-patient variance and not for within-practice variance (2). Ignoring within-practice variance could lead to less precise results and wider confidence intervals (4).

The models were assessed on the proportional hazards assumption and discrimination using the concordance index (29). For the sensitivity analyses, stage three was repeated on patients with complete EHR, and on restricting the controls only to patients who were never prescribed statins (on imputed datasets). All statistical analyses were carried out in R version 3.5.0, except for the QRISK2 score calculation in JAVA version 10.

References

1. van Houwelingen HC. Dynamic Prediction by Landmarking in Event History Analysis. Scand J Stat. 2007 Mar;34(1):70–85.
2. van Houwelingen HC, Putter H. Dynamic Prediction in Clinical Survival Analysis. Dynamic Prediction in Clinical Survival Analysis. CRC Press; 2011.
3. Rubin D. Multiple Imputation for Nonresponse in Surveys. Rubin DB, editor. Hoboken, NJ, USA: John Wiley & Sons, Inc.; 1987. (Wiley Series in Probability and Statistics).
4. Chuang JH, Hripcsak G, Heitjan DF. Design and analysis of controlled trials in naturally clustered environments: Implications for medical informatics. J Am Med Informatics Assoc. 2002;9(3):230–8.

S1 Table. Coding of medical history.

Variable	Description[a]	Coding[b]
Alcohol	Alcohol consumer status	Non-current / current
Aspirin	Aspirin prescription (BNF chapter 2.9.1)	No / yes
BMI	Body mass index calculated as: (weight in kg)/(height in m)²	under/normal weight: <25, overweight: 25-30, obese: ≥30
Cardiac risk	Based on QRISK2 risk score (10-year risk of first cardiac event) and diagnosis of cardiovascular disease (CVD: coronary heart disease and cerebrovascular disease but not peripheral vascular disease)	QRISK2 of 20-40% / QRISK2 ≥40% or CVD diagnosis / QRISK2<20%
CKD	Diagnosis of chronic kidney disease at stage 3-5 (GFR<15, (ICD-10 N18.3-5)	No / yes
Deprivation	Townsend deprivation index, which is based on unemployment, non-car ownership, non-home ownership, and household overcrowding.	Quintiles where 1 is least deprived and 5 most deprived
Diabetes	Diagnosis of diabetes mellitus type 2 (ICD-10 E10-14)	No / yes
HCL	Diagnosis of hypercholesterolaemia (ICD-10 E78.0-5) or total cholesterol reading greater than 5mmol/L	No / yes
Hypertension	Based on diagnosis (ICD-10 I10) and prescription of antihypertensive treatment (BNF chapters 2.4, 2.5.5 and 2.6.2: beta-adrenoceptor blocking drugs, thiazides and related diuretics, adrenergic neurone blocking drugs, alpha-adrenoceptor blocking drugs, angiotensin-converting enzyme inhibitors, angiotensin-II receptor antagonists, centrally acting antihypertensive drugs, drugs affecting the renin-angiotensin system, drugs related to hypertension and heart failure, renin inhibitors, vasodilator antihypertensive drugs, and calcium-channel blockers)	No diagnosis / diagnosed & treated / diagnosed & not treated
Sex	Gender	Women / men
Smoking	Smoking status	Non / ex / current
Statins	Current prescription (BNF chapter 2.12), which include: atorvastatin, cerivastatin, fluvastatin, pravastatin, rosuvastatin, and simvastatin	No / yes
SBP	Systolic blood pressure measured as mm Hg	
YoB	Year of birth category	1930-35 / 1936-40

[a] Latest reading at landmark age. [b] First category functioned as the reference group.

S2 Table. Characteristics of patients with complete and incomplete[a] health records.

	Records	Year of birth	Size	Death/1,000 Person-Years	Male (%)	Statins (%)	CVD (%)
Age 60	Complete	1936-40	30,012	4.64	12,784 (43%)	NA	NA
		1930-35	10,824	2.82	4616 (43%)	NA	NA
	Incomplete	1936-40	38,182	5.82	19,245 (50%)	NA	NA
		1930-35	31,225	8.58	14,202 (45%)	NA	NA
Age 65	Complete	1936-40	34,151	5.09	14,991 (44%)	4822 (14%)	2302 (7%)
		1930-35	17,034	4.56	7447 (44%)	833 (5%)	1356 (8%)
	Incomplete	1936-40	28,931	4.39	14,402 (50%)	1579 (5%)	1432 (5%)
		1930-35	24,265	6.66	10,988 (45%)	384 (2%)	1268 (5%)
Age 70	Complete	1936-40	36,008	4.41	16,028 (45%)	13,571 (38%)	4981 (14%)
		1930-35	18,530	4.81	8153 (44%)	4184 (23%)	2996 (16%)
	Incomplete	1936-40	18,896	2.61	9074 (48%)	3322 (18%)	1941 (10%)
		1930-35	19,401	5.30	8492 (44%)	1663 (9%)	2179 (11%)
Age 75	Complete	1936-40	35,123	2.29	15,654 (45%)	16,980 (48%)	7145 (20%)
		1930-35	19,642	4.23	8558 (44%)	8944 (46%)	4683 (24%)
	Incomplete	1936-40	11,329	1.08	5181 (46%)	3230 (29%)	1814 (16%)
		1930-35	12,634	3.24	5182 (41%)	2487 (20%)	2119 (17%)
Age 80	Complete	1936-37	4617	0.07	2044 (44%)	2392 (52%)	1304 (28%)
		1930-35	17,962	2.21	7699 (43%)	9065 (50%)	5240 (29%)
	Incomplete	1936-37	1111	0.02	471 (42%)	430 (39%)	265 (24%)
		1930-35	7477	1.39	2875 (38%)	2065 (28%)	1648 (22%)
Age 85	Complete	1930-32	3002	0.16	1271 (42%)	1421 (47%)	1052 (35%)
	Incomplete	1930-32	1099	0.08	351 (32%)	416 (38%)	276 (25%)

[a]Missing record in systolic blood pressure, body mass index, alcohol consumer status or smoking status.

S1 Figure. Distribution of observed and imputed values of variables with missing data at baseline.

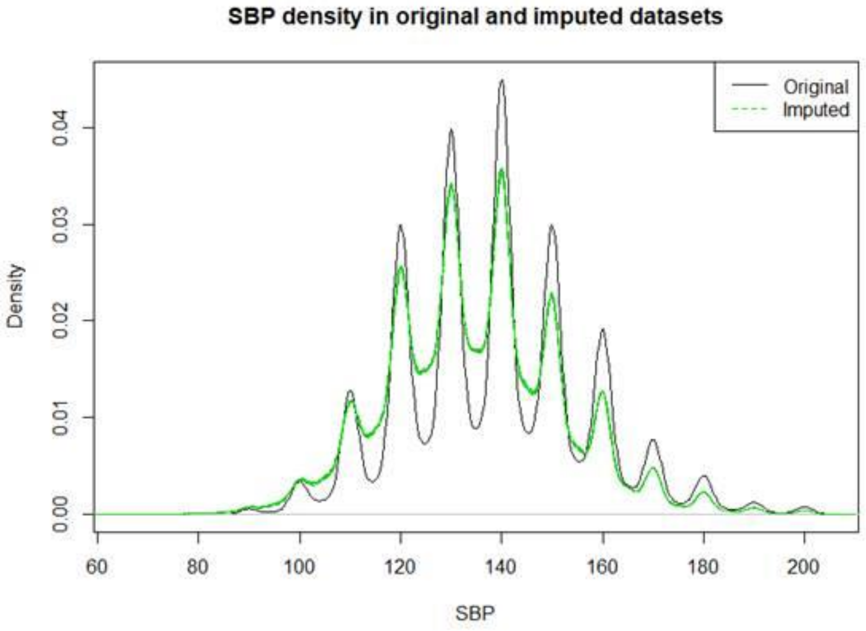

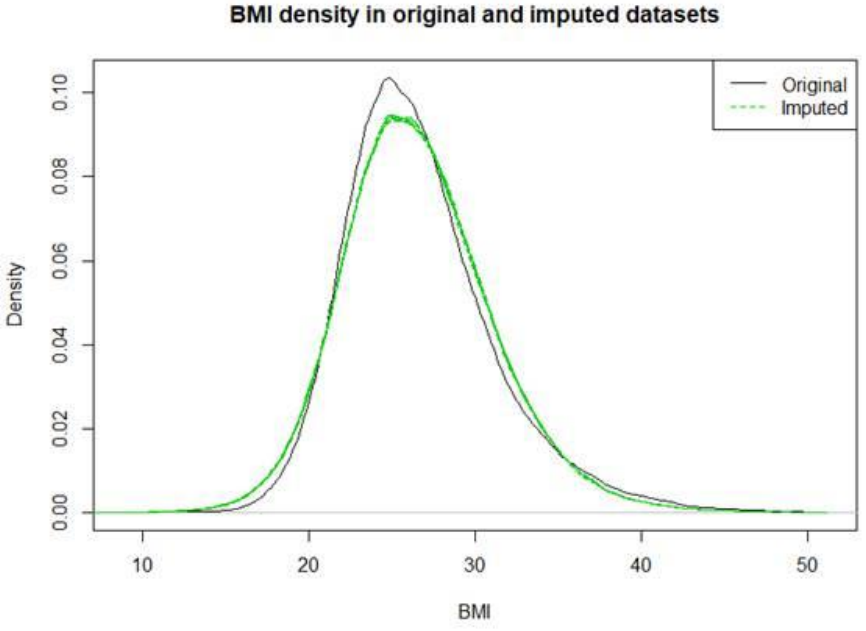

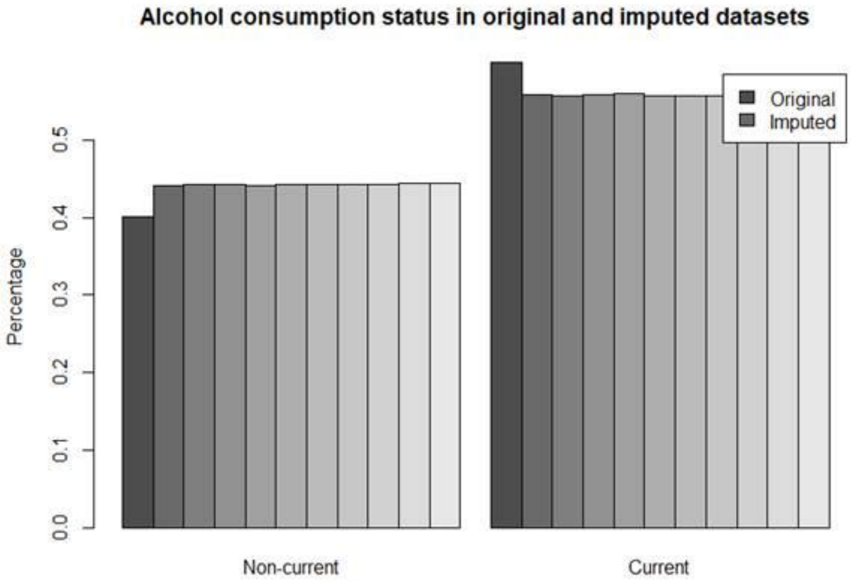
Alcohol consumption status in original and imputed datasets
Percentage
0.0
0.1
0.2
0.3
0.4
0.5
Non-current
Current
Original
Imputed

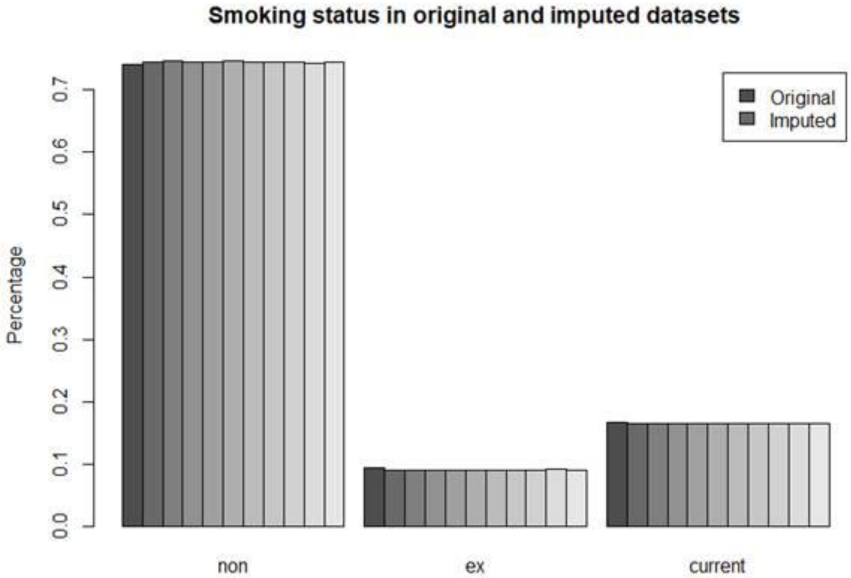
Smoking status in original and imputed datasets
Percentage
0.0
0.1
0.2
0.3
0.4
0.5
0.6
0.7
non
ex
current
Original
Imputed

S3 Table. Years of follow-up by cardiac risk and landmark age.

	QRISK2<20%			QRISK2: 20-40%			QRISK2≥40% or CVD		
	Mean	Min	Max	Mean	Min	Max	Mean	Min	Max
Age 60	16.6	<1	26.5	14.3	<1	26.5	11.4	1.6	21.5
Age 65	12.6	<1	21.5	11.0	<1	21.5	11.6	<1	21.5
Age 70	9.0	<1	16.5	8.2	<1	16.5	8.0	<1	16.5
Age 75	4.9	<1	11.5	4.7	<1	11.5	4.4	<1	11.5
Age 80	NA	NA	NA	2.7	<1	6.5	2.5	<1	6.5
Age 85	NA	NA	NA	0.9	<1	1.5	0.8	<1	1.5

S4 Table. Cardiac risk[a] by sex and landmark age.

	Age 65	Age 70	Age 75	Age 80	Age 85
Women					
QRISK2<20%	51,819 (92%)	35,337 (69%)	7127 (16%)	0 (0%)	0 (0%)
QRISK2:20-40%	2122 (4%)	10,149 (20%)	27,672 (63%)	10,564 (58%)	221 (9%)
QRISK2≥40% or CVD	2612 (5%)	5602 (11%)	9354 (21%)	7514 (42%)	2258 (91%)
Total (100%)	56,553	51,088	44,153	18,078	2479
Men					
QRISK2<20%	32,561 (68%)	10,019 (24%)	0 (0%)	0 (0%)	0 (0%)
QRISK2:20-40%	11,288 (24%)	23,587 (56%)	21,463 (62%)	4347 (33%)	31 (2%)
QRISK2≥40% or CVD	3979 (8%)	8141 (20%)	13,112 (38%)	8742 (67%)	1591 (98%)
Total (100%)	47,828	41,747	34,575	13,089	1622

[a]Mean across ten imputed datasets.

Gitsels LA, *et al. Fam Med Com Health* 2021; 9:e000780. doi: 10.1136/fmch-2020-000780

S2 Figure. Cumulative years[a] of statin therapy by the landmark age in patients with current statin prescription by year of birth.

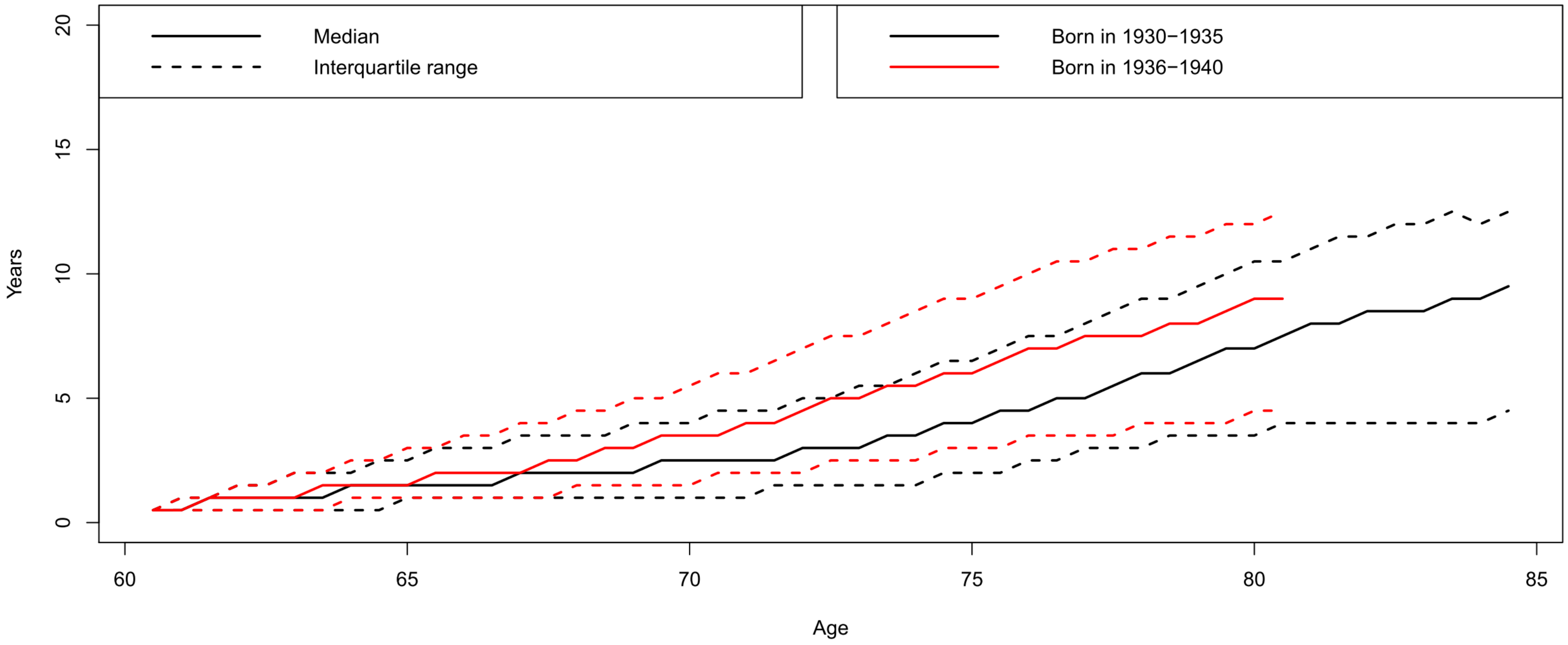

[a]Cumulative years of statin therapy was defined from first prescription until landmark *s* in patients with current statin prescription at landmark *s*.

S3 Figure. Proportion of high adherence[a] to statin therapy in patients with a current statin prescription by year of birth.

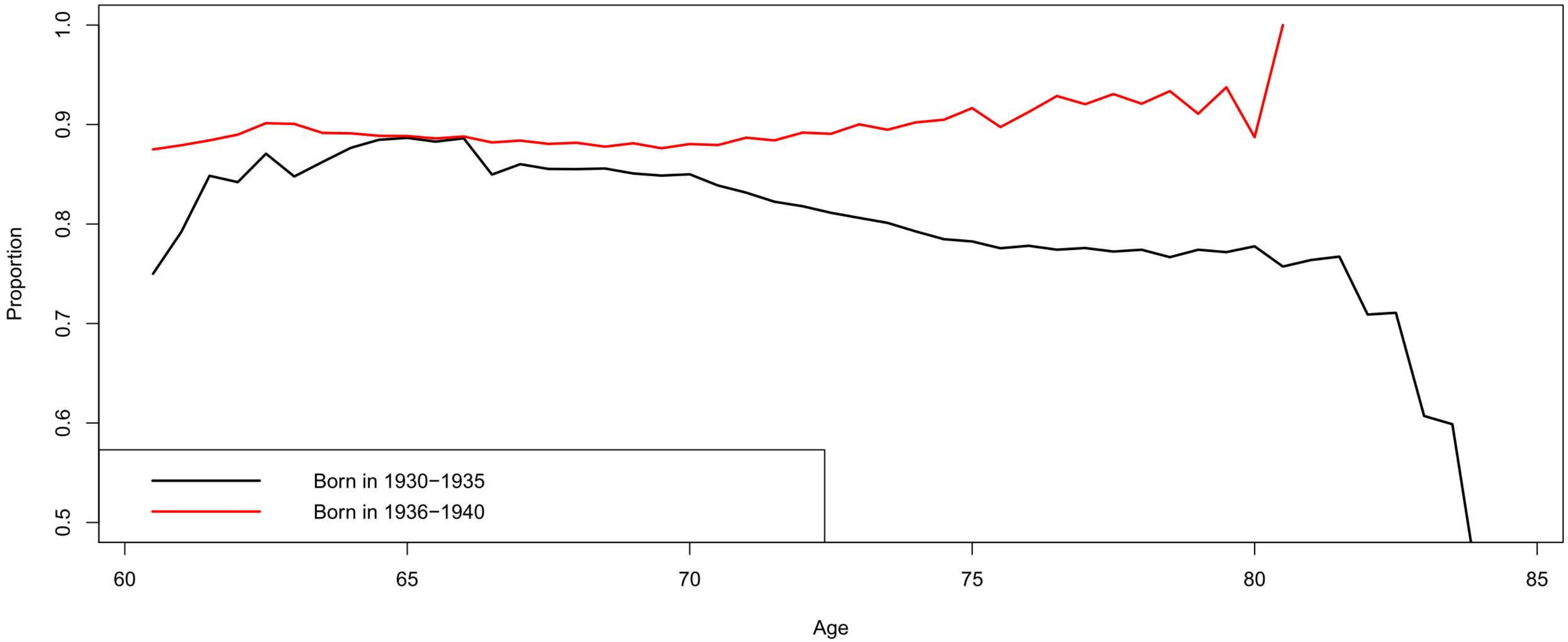

[a]High adherence to statin therapy was defined as statin prescription at least 75% of the time from landmark *s* until end of follow-up in patients with a current statin prescription at *s*.

S4 Figure. Average cumulative years[a] of statin therapy by the landmark age in patients with no current statin prescription by year of birth.

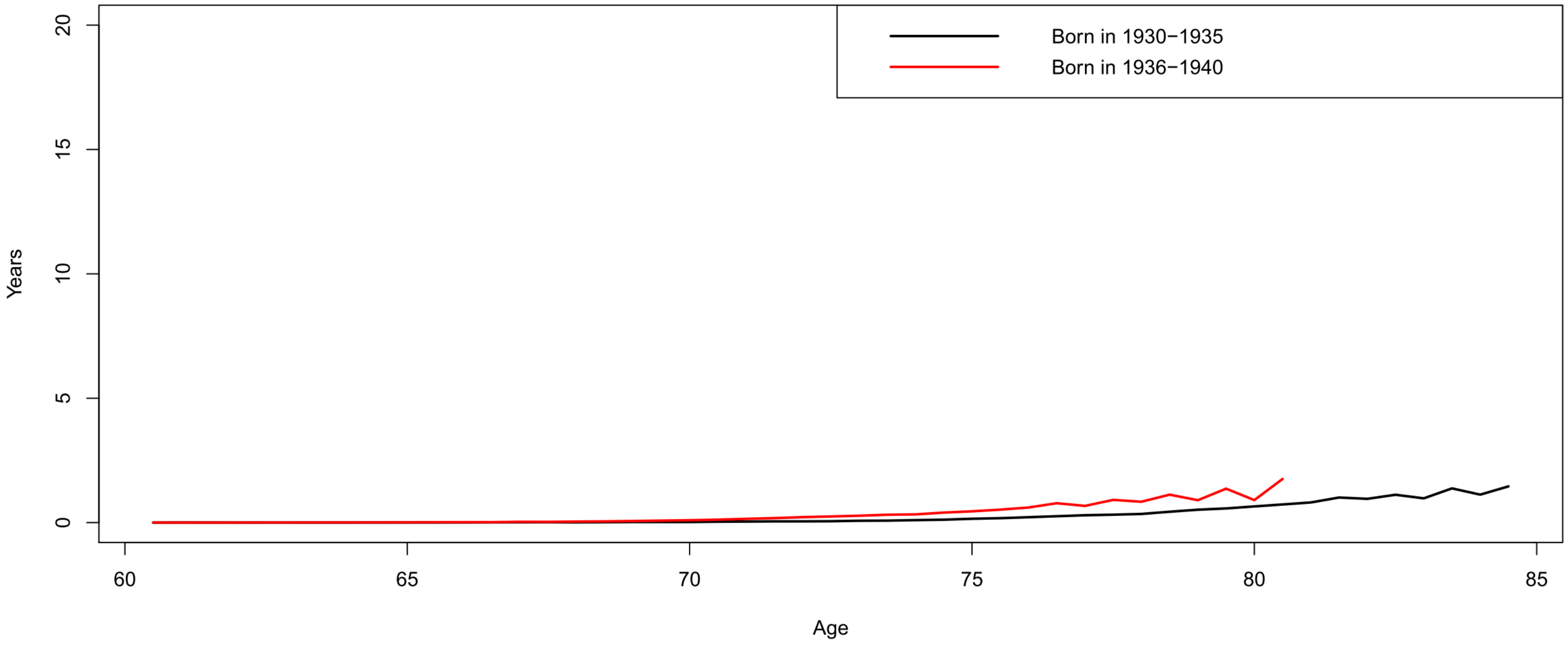

[a]Cumulative years of statin therapy was defined from first prescription until landmark *s-1* in patients with no current statin prescription at landmark *s* and was zero for patients who never had a statin prescription until landmark *s-1*.

S5 Table. Hazards of all-cause mortality from landmark model fitted on imputed datasets.

Covariate	Category	Age 65 HR[a] (95%CI) (n=104,375)	Age 70 HR[a] (95%CI) (n=92,829)	Age 75 HR[a] (95%CI) (n=78,721)	Age 80 HR[a] (95%CI) (n=31,166)	Age 85 HR[a] (95%CI) (n=4101)
Statins[b] - unadjusted 25-yr prediction	No prescription	1	1	1	1	1
	Prescription	1.21 (1.15-1.27)	1.10 (1.07-1.13)	1.01 (0.98-1.04)	0.91 (0.87-0.96)	0.80 (0.70-0.91)
Statins[b] 5-yr prediction	No prescription	1	1	1	1	1
	Prescription & YoB=1930-35	0.82 (0.68-0.99)	0.84 (0.78-0.90)	0.77 (0.73-0.81)	0.73 (0.68-0.77)	0.83 (0.68-1.01)
	Prescription & YoB=1936-40	0.75 (0.68-0.82)	0.68 (0.64-0.72)	0.67 (0.63-0.71)	0.61 (0.50-0.73)	0.43 (0.20-0.93)
Statins[b] 10-yr prediction	No prescription	1	1	1	1	1
	Prescription & YoB=1930-35	0.87 (0.76-0.99)	0.86 (0.82-0.91)	0.78 (0.74-0.82)	0.73 (0.69-0.78)	0.83 (0.70-0.99)
	Prescription & YoB=1936-40	0.76 (0.71-0.81)	0.71 (0.68-0.75)	0.68 (0.65-0.72)	0.63 (0.53-0.73)	0.54 (0.33-0.92)
Statins[b] 25-yr prediction	No prescription	1	1	1	1	1
	Prescription & YoB=1930-35	0.92 (0.83-1.01)	0.87 (0.82-0.91)	0.79 (0.75-0.83)	0.74 (0.70-0.78)	0.76 (0.65-0.89)
	Prescription & YoB=1936-40	0.80 (0.75-0.85)	0.73 (0.69-0.76)	0.68 (0.65-0.73)	0.63 (0.55-0.74)	0.65 (0.13-3.27)
Cardiac risk	QRISK2: 20-40%	1	1	1	1	1
	QRISK2>=40% or CVD diagnosis	1.27 (1.20 - 1.34)	1.41 (1.35 - 1.46)	1.43 (1.37 - 1.49)	1.34 (1.26-1.42)	1.13 (0.95-1.33)
	QRISK2<20%	0.91 (0.87 - 0.94)	0.90 (0.87 - 0.93)	0.98 (0.92 - 1.04)	NA	NA
Sex	Men	1	1	1	1	1
	Women	0.75 (0.73 - 0.77)	0.77 (0.75 - 0.79)	0.77 (0.74 - 0.80)	0.77 (0.73 - 0.82)	0.78 (0.68 - 0.90)
Deprivation	1st quintile	1	1	1	1	1
	2nd quintile	1.07 (1.03 - 1.12)	1.09 (1.04 - 1.14)	1.09 (1.03 - 1.15)	1.03 (0.95 - 1.12)	0.90 (0.75 - 1.07)

Covariate	Category	Age 65 HR[a] (95%CI) (n=104,375)	Age 70 HR[a] (95%CI) (n=92,829)	Age 75 HR[a] (95%CI) (n=78,721)	Age 80 HR[a] (95%CI) (n=31,166)	Age 85 HR[a] (95%CI) (n=4101)
	3rd quintile	1.16 (1.11 - 1.21)	1.17 (1.12 - 1.22	1.15 (1.09 - 1.22)	1.08 (0.99 - 1.17)	0.92 (0.76 - 1.11)
	4th quintile	1.26 (1.20 - 1.31)	1.27 (1.22 - 1.33)	1.28 (1.20 - 1.34)	1.19 (1.10 - 1.30)	1.00 (0.83 - 1.20)
	5th quintile (most deprived)	1.44 (1.38 - 1.51)	1.39 (1.32 - 1.46)	1.31 (1.24 - 1.39)	1.22 (1.11 - 1.33)	1.12 (0.91 - 1.37)
CKD	No diagnosis	1	1	1	1	1
	Diagnosis	1.35 (1.07 - 1.70)	1.34 (1.26 - 1.44)	1.27 (1.21 - 1.33)	1.24 (1.17 - 1.32)	1.39 (1.18 - 1.64)
Diabetes	No diagnosis	1	1	1	1	1
	Diagnosis	1.60 (1.52 - 1.68)	1.43 (1.37 - 1.50)	1.32 (1.26 - 1.39)	1.25 (1.17 - 1.34)	1.20 (1.01 - 1.42)
Hypertension	No diagnosis	1	1	1	1	1
	Diagnosed & treated	1.15 (1.11 - 1.19)	1.06 (1.02 - 1.09)	0.98 (0.94 - 1.02)	0.90 (0.85-0.95)	0.81 (0.70 - 0.94)
	Diagnosed & not treated	1.05 (1.02 - 1.10)	1.06 (1.01 - 1.10)	1.09 (1.03 - 1.15)	1.21 (1.12-1.32)	1.51 (1.24 - 1.84)
Aspirin	No prescription	1	1	1	1	1
	Prescription	1.17 (1.12 - 1.22)	1.08 (1.04 - 1.12)	1.09 (1.05 - 1.14)	1.13 (1.07 - 1.20)	1.12 (0.96 - 1.30)
HCL	No diagnosis	1	1	1	1	1
	Diagnosis	0.83 (0.79 - 0.88)	0.87 (0.83- 0.91)	0.91 (0.86-0.96)	0.95 (0.88 - 1.02)	0.98 (0.82-1.19)
BMI	Normal weight	1	1	1	1	1
	Overweight	0.94 (0.90-0.98)	0.90 (0.87-0.93)	0.87 (0.83-0.90)	0.86 (0.81 - 0.91)	0.91 (0.78 - 1.06)
	Obese	1.07 (1.02-1.13)	1.02 (0.98-1.07)	0.96 (0.91- 1.01)	0.90 (0.83 - 0.97)	0.86 (0.70 - 1.04)
Alcohol	Non-current	1	1	1	1	1
	Current	0.87 (0.84-0.89)	0.83 (0.80 -0.86)	0.78 (0.75-0.81)	0.71 (0.67 - 0.75)	0.65 (0.56 - 0.74)
Smoking	Non	1	1	1	1	1
	Ex	1.45 (1.40 - 1.51)	1.45 (1.40 - 1.50)	1.43 (1.38 - 1.49)	1.37 (1.29 - 1.45)	1.24 (1.08 - 1.42)
	Current	2.34 (2.26 - 2.43)	2.27 (2.19 - 2.36)	2.13 (2.03 - 2.23)	1.85 (1.70 - 2.02)	1.42 (1.12 - 1.78)

[a]All hazard ratios were adjusted by the factors listed in this table except for 'statins - unadjusted'. [b]Prescription at landmark age.

S5 Figure. Incidence of diabetes in patients with or without prior statin prescription by the landmark age.

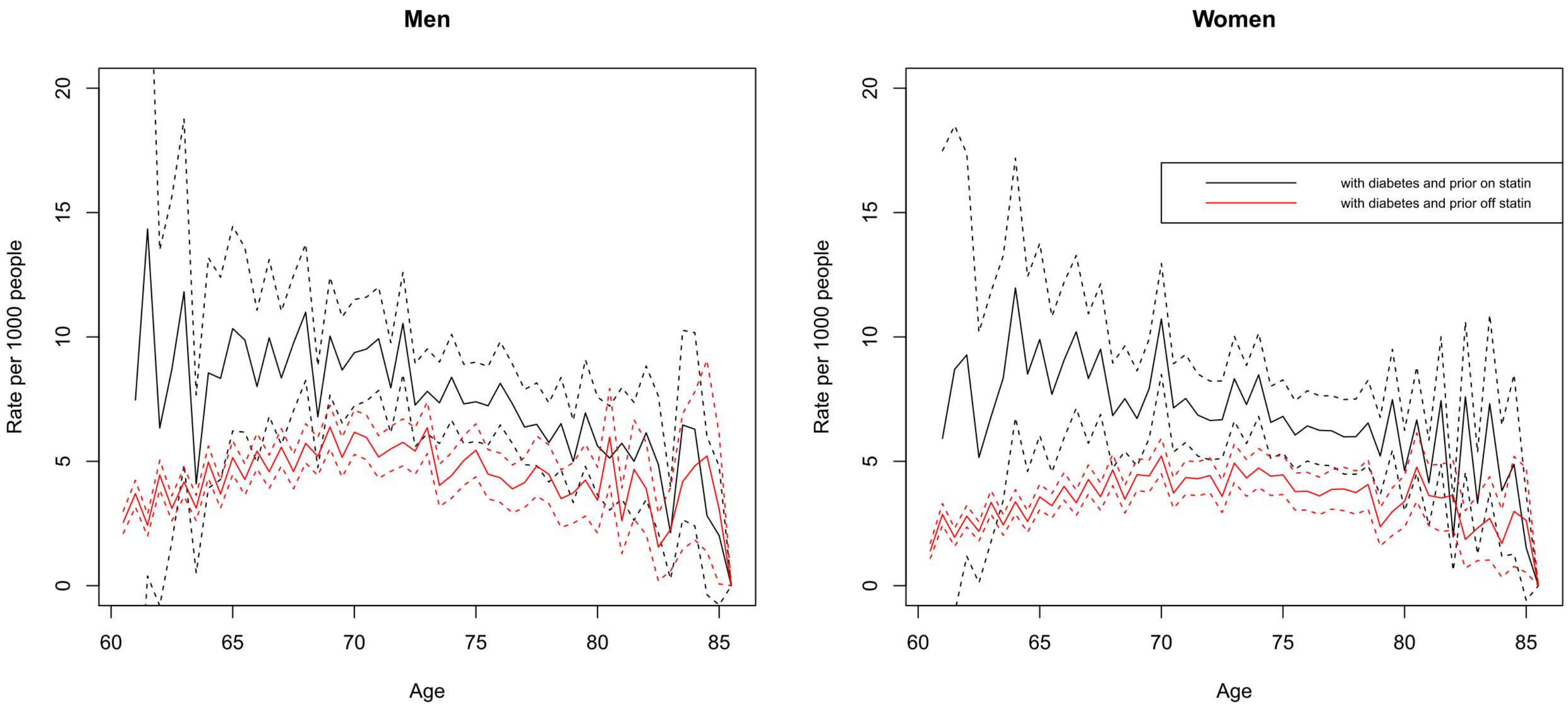

S6 Table. Hazards of all-cause mortality from landmark model fitted on complete cases.

Covariate	Category	Age 65 HR[a] (95%CI) (n=51,185)	Age 70 HR[a] (95%CI) (n=54,538)	Age 75 HR[a] (95%CI) (n=54,765)	Age 80 HR[a] (95%CI) (n=22,579)	Age 85 HR[a] (95%CI) (n=3002)
Statins[b] - unadjusted	No prescription	1	1	1	1	1
	Prescription	1.17 (1.10- 1.26)	1.13 (1.08-1.19)	1.09 (1.03-1.16)	1.01 (0.91-1.12)	0.85 (0.62-1.18)
Statins[b]	No prescription	1	1	1	1	1
	Prescription & YoB=1930-35	0.98 (0.84 - 1.13)	0.91 (0.83 - 0.98)	0.84 (0.78 - 0.91)	0.78 (0.69 - 0.89)	0.72 (0.45 - 1.04)
	Prescription & YoB=1936-40	0.77 (0.71 - 0.84)	0.73 (0.68 - 0.78)	0.71 (0.65 -0.78)	0.61 (0.48 - 0.77)	0.41 (0.18 - 0.92)
Cardiac risk	QRISK2: 20-40%	1	1	1	1	1
	QRISK2>=40% or CVD diagnosis	1.34 (1.23 - 1.45)	1.43 (1.34 - 1.52)	1.36 (1.22 - 1.43)	1.25 (1.09 - 1.42)	1.38 (0.89 - 2.15)
	QRISK2<20%	0.90 (0.85 - 0.95)	0.89 (0.84 - 0.94)	0.91 (0.81 - 1.03)	NA	NA
Sex	Men	1	1	1	1	1
	Women	0.73 (0.69 - 0.77)	0.75 (0.71 - 0.79)	0.75 (0.69 - 0.80)	0.72 (0.63 - 0.82)	0.69 (0.49 - 0.96)
Deprivation	1st quintile	1	1	1	1	1
	2nd quintile	1.03 (0.96 - 1.11)	1.06 (0.98 - 1.15)	1.05 (0.96 - 1.17)	0.99 (0.83 - 1.18)	0.83 (0.52 - 1.31)
	3rd quintile	1.17 (1.09 - 1.26)	1.21 (1.12 - 1.31)	1.22 (1.11 - 1.35)	1.16 (0.97 - 1.38)	0.97 (0.62 - 1.53)
	4th quintile	1.28 (1.19 - 1.37)	1.31 (1.21 - 1.42)	1.35 (1.22 - 1.49)	1.31 (1.10 - 1.57)	1.15 (0.73 - 1.80)
	5th quintile (most deprived)	1.50 (1.38 - 1.62)	1.46 (1.34 - 1.59)	1.41 (1.26 - 1.57)	1.34 (1.10 - 1.63)	1.24 (0.75 - 2.06)
CKD	No diagnosis	1	1	1	1	1
	Diagnosis	1.39 (1.04 - 1.86)	1.38 (1.26 - 1.52)	1.29 (1.20 - 1.40)	1.26 (1.12 - 1.43)	1.45 (0.97 - 2.18)
Diabetes	No diagnosis	1	1	1	1	1
	Diagnosis	1.66 (1.54 - 1.80)	1.47 (1.37 - 1.58)	1.35 (1.23 - 1.47)	1.30 (1.12 - 1.51)	1.36 (0.90 - 2.05)
Hypertension	No diagnosis	1	1	1	1	1
	Diagnosed & treated	1.30 (1.23 - 1.37)	1.22 (1.15 - 1.29)	1.17 (1.08 - 1.26)	1.13 (0.98 - 1.30)	1.06 (0.73 - 1.54)

Covariate	Category	Age 65 HR[a] (95%CI) (n=51,185)	Age 70 HR[a] (95%CI) (n=54,538)	Age 75 HR[a] (95%CI) (n=54,765)	Age 80 HR[a] (95%CI) (n=22,579)	Age 85 HR[a] (95%CI) (n=3002)
	Diagnosed & not treated	1.20 (1.13 - 1.27)	1.19 (1.11 - 1.29)	1.20 (1.07 - 1.34)	1.22 (1.00 - 1.49	1.28 (0.76 - 2.14)
Aspirin	No prescription	1	1	1	1	1
	Prescription	1.09 (1.02 - 1.16)	1.04 (0.98 - 1.10)	1.12 (1.04 - 1.20)	1.11 (0.99 - 1.24)	0.82 (0.58 - 1.18)
HCL	No diagnosis	1	1	1	1	1
	Diagnosis	0.85 (0.79 - 0.92)	0.87 (0.81 - 0.93)	0.89 (0.82 - 0.97)	0.93 (0.80 - 1.07)	1.00 (0.69 - 1.47)
BMI	Normal weight	1	1	1	1	1
	Overweight	0.89 (0.84 - 0.94)	0.83 (0.79 - 0.88)	0.79 (0.73 - 0.85)	0.85 (0.75 - 0.96)	1.15 (0.82 - 1.61)
	Obese	1.06 (0.99 - 1.13)	0.95 0.89 - 1.02)	0.87 (0.80 - 0.95)	0.88 (0.75 - 1.02)	1.06 (0.70 - 1.60)
Alcohol	Non-current	1	1	1	1	1
	Current	0.92 (0.88 - 0.98)	0.89 (0.84 - 0.94)	0.85 (0.79 - 0.90)	0.77 (0.69 - 0.87)	0.64 (0.47 - 0.88)
Smoking	Non	1	1	1	1	1
	Ex	1.56 (1.47 - 1.65)	1.58 (1.49 - 1.67)	1.57 (1.46 - 1.68)	1.59 (1.40 - 1.80)	1.70 (1.20 - 2.39)
	Current	2.49 (2.35 - 2.63	2.48 (2.32 - 2.65)	2.43 (2.22 - 2.65)	2.22 (1.83 - 2.67)	1.78 (1.05 - 3.02)

[a]All hazard ratios are adjusted by the factors listed in this table except for 'statins - unadjusted'. [b]Prescription at landmark age.

S6 Figure. Sensitivity analysis: hazards of all-cause mortality associated with current statin prescription excluding controls with future statin prescription fitted on imputed datasets.

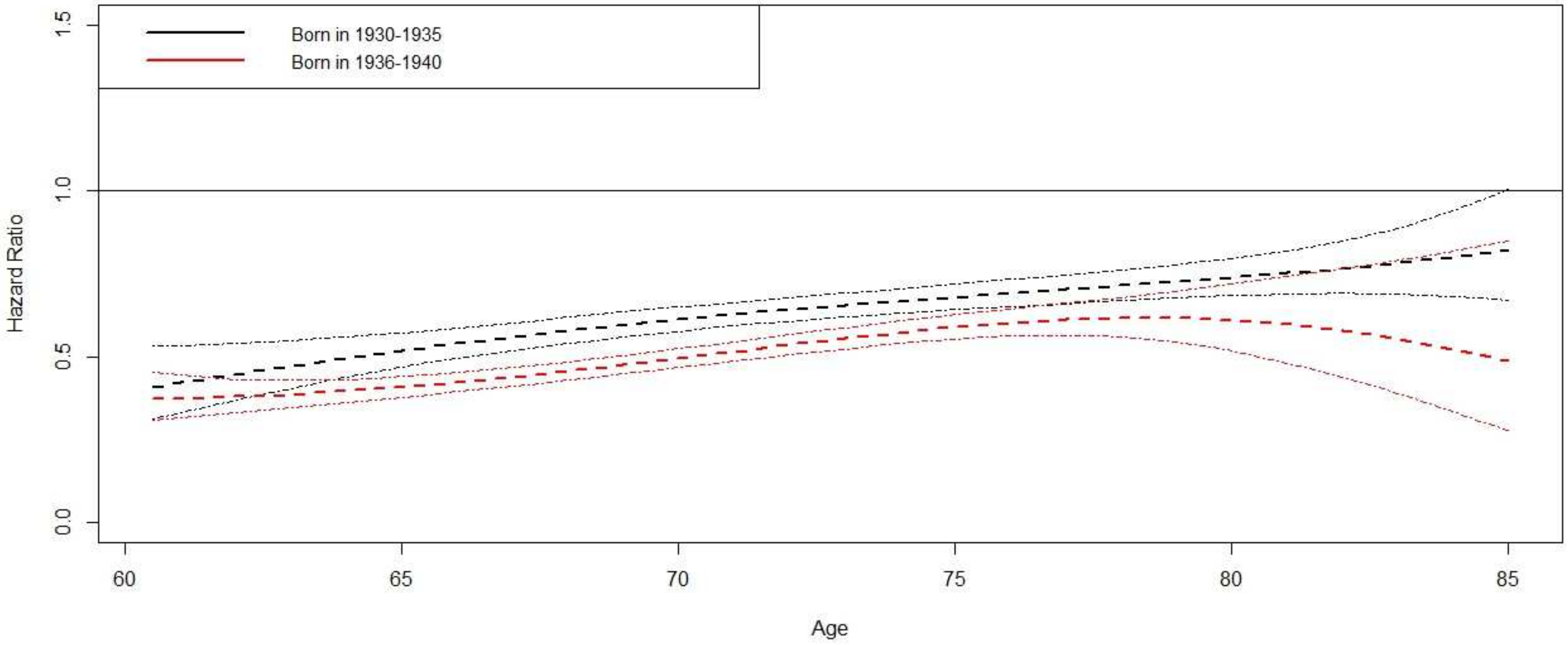

Supplement: Supplementary data [file fmch-2020-000780supp001.pdf]
